# AID modulates carcinogenesis network via DNA demethylation in bladder urothelial cell carcinoma

**DOI:** 10.1038/s41419-019-1472-x

**Published:** 2019-03-15

**Authors:** Haoyong Li, Qi Li, Zhe Ma, Zhiyan Zhou, Jinfeng Fan, Yingxia Jin, Yaoxi Wu, Fan Cheng, Peiyu Liang

**Affiliations:** 1 Department of Urology, Renmin Hospital of Wuhan University, Wuhan, Hubei Province China; 2 Department of Urology, the First Affiliated Hospital of Hainan Medical College, Haikou, Hainan Province China; 3 Central Laboratory, Renmin Hospital of Wuhan University, Wuhan, Hubei Province China

## Abstract

Bladder cancer is one of the most common malignant diseases in the urinary system, with poor survival after metastasis. Activation-induced cytidine deaminase (AID), a versatile enzyme involved in antibody diversification, is an oncogenic gene that induces somatic hypermutation and class-switch recombination (CSR). However, the contribution of AID-mediated DNA demethylation to bladder urothelial cell carcinoma (BUCC) remains unclear. Herein, we evaluated the impact on BUCC caused by AID and explored the gene network downstream of AID by using a proteomic approach. Lentiviral vector containing AID-specific shRNA significantly reduced AID expression in T24 and 5637 cells. Silencing AID expression remarkably inhibited tumour malignancies, including cell proliferation, invasion and migration. We used Isobaric tags for relative and absolute quantitation (iTRAQ)-based proteomics analysis technology to study the underpinning mechanism in monoclonal T24 cells, with or without AID knockdown. Among the 6452 proteins identified, 99 and 142 proteins in shAICDA-T24 cells were significantly up- or downregulated, respectively (1.2-fold change) compared with the NC-T24 control. After a pipeline of bioinformatics analyses, we identified three tumour-associated factors, namely, matrix metallopeptidase 14 (MMP14), C–X–C motif chemokine ligand 12 and wntless Wnt ligand secretion mediator, which were further confirmed in human BUCC tissues. Nonetheless, only MMP14 was sensitive to the DNA demethylation molecule 5-aza-2’-deoxycytidine (5-azadC; 5 μM), which reversed the inhibition of carcinogenesis by AID silence in T24 and 5637 cells. Overall, AID is an oncogene that mediates tumourigenesis via DNA demethylation. Our findings provide novel insights into the clinical treatment for BUCC.

## Introduction

Bladder urothelial cell carcinoma (BUCC) is one of the most common malignant diseases in urinary systems and is the fourth most common cancer in men of China^1^. The incidence of bladder cancer has gradually increased in the past decade. In the clinical treatment of BUCC, various factors, including HER-2, H-ras, Bcl-2 and FGFR3, are regarded as the therapeutic target. However, the success of clinical treatment is limited after metastasis occurs. Therefore, searching for the progression factors of BUCC is critical to improve the clinical treatment of the disease.

Activation-induced cytidine deaminase (AID), a member of the deaminase family, can lead to dU:dG mismatches by dC to dU deamination^2^. This enzyme is usually expressed in the germinal centre B cells (GCBs) and regulates the secondary antibody diversification through somatic hypermutation (SHM; point mutation in IgV) and class-switch recombination (CSR; double-strand break in IgH)^3^, leading to affinity maturation and antibody isotype conversion (from IgM to IgA, IgE and IgG), respectively. Furthermore, AID is also associated with the loss of DNA methylation^4^. Methylation modification usually occurs in CpG islands, which are mainly located in the regions of promoter and exon, participating in epigenetic mechanisms by inhibiting the initiation of transcription. AID can trigger mismatch repair and base excision repair by the deamination of 5-methylcytosine^5,6^. Consequently, dmC is replaced by dC, and demethylation is achieved. Interestingly, demethylation by AID is consistent with SHM and CSR, suggesting that the deamination of dmC is also required for antibody diversification, such as dC^7^. However, the specific mechanism of AID-induced demethylation is poorly understood.

The regulation of AID is complex, with multi-level and multiple factors. Considering the lack of protection against heat-shock proteins, the proteasome may be more likely to be degraded in the nucleus than in the cytoplasm;^8^ thus, AID is more unstable through an effective ubiquitination in the nucleus^9^. Therefore, the shuttle is a key way of management between the nucleus and cytoplasm^10^. Additionally, the phosphorylation of amino acid residues, including S38, T140 and S3, is associated with the regulation of AID^11–13^. Many factors, including CD40 ligand, NF-κB, PAX5, E2f, STAT6 and Smad3/4, are also involved in the regulation of AID^14–17^. Scholars recently discovered that AID is not only limited to GCBs but also to multiple organs. This trait associates AID with various diseases during dysregulation, but is mostly noted in malignant diseases. AID is closely related to tumourigenesis, including leukaemia^18^, lymphoma^19^, lung cancer^20^, skin cancer^21^ and oesophageal adenocarcinoma^22^. Furthermore, AID-induced demethylation is also involved in the expression of tumour progression factors^23^. According to functional characteristics, AID is upregulated during inflammation, thereby participating in cancer-related diseases, such as *Helicobacter pylori*-induced gastritis and viral hepatitis^24,25^. Hence, AID establishes a connection between inflammation and tumour, and the upregulation of AID by inflammation is necessary for epithelial-to-mesenchymal transition (EMT) in mammary cancer^23^. Overall, AID is a key enzyme in the human immune system with strict management, but is closely associated with the occurrence and progression of tumour during dysregulation. However, the progression mechanism of AID-related malignancy is still poorly understood.

In classical immunology, only B lymphocytes can produce immunoglobulins. Herein, we detected IgG expression in BUCC cells (BIU-87 and T24)^26^. We also found that inhibiting the expression of IgG1 derived from cancer cells could significantly inhibit cell migration and proliferation in human BUCC 5637 cells by knockdown IGHG1 gene and could also promote cell apoptosis. According to the existing research on AID and its molecular function (MF), AID may play a positive role in the progression of BUCC.

This study aimed to screen AID-related progression factors in BUCC T24 cells by using iTRAQ-based proteomic methods and bioinformatics analysis and to elucidate the mechanism of regulation between them. Herein, we screened out three factors, namely, matrix metallopeptidase 14 (MMP14), C–X–C motif chemokine ligand 12 (CXCL12/SDF-1) and wntless Wnt ligand secretion mediator (WLS/GPR177), by Cytoscape software through Maximal Clique Centrality (MCC) algorithm. These factors indicate that AID is related to the migration and invasion of T24 cells. We confirmed that AID enhances the invasiveness and participates in the EMT of BUCC cells by demethylation. In summary, AID is widely involved in the development of BUCC and may become a potential therapeutic target for clinical treatment.

## Results

### AID expression is upregulated in human BUCC tissues and cell lines

**Fig. 1:**
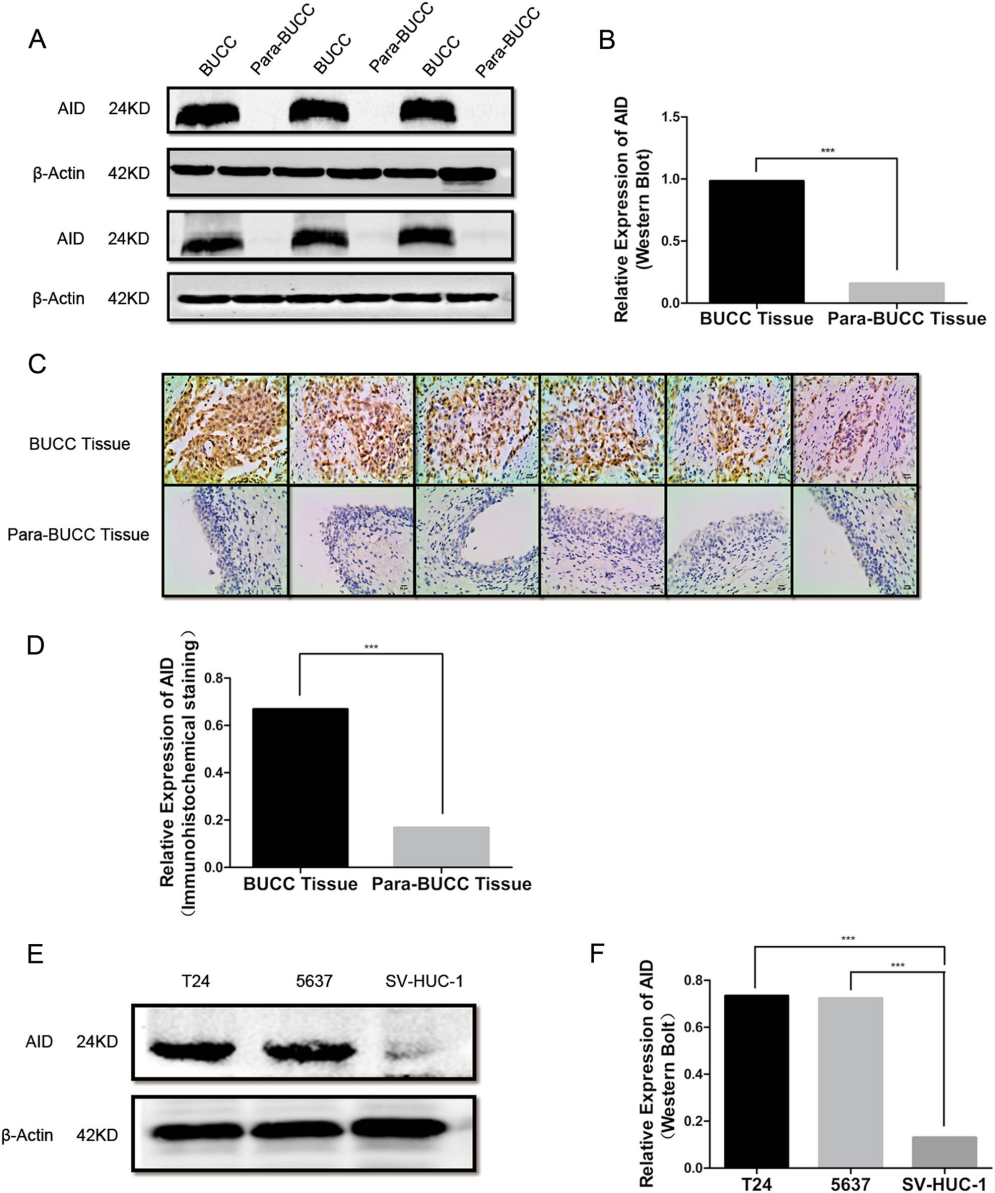
AID expression in human bladder carcinoma/para-carcinoma tissues and BUCC cells. **a**–**d** AID was detected by western blot and IHC in human BUCC/para-BUCC tissues. Original magnification: **c**, × 200, calibration bar at 50 μm. The expression of AID in human BUCC tissues was more than that in para-BUCC tissues (BC: 0.98 ± 0.19, PC: 0.16 ± 0.05, *P* < 0.05). **d**, **e** The similar results found by western blot in BUCC cell lines; the relative expression of AID in bladder cancer cells T24 and 5637 was significantly more than that in SV-HUC-1 cell (T24: 0.73 ± 0.07, 5637: 0.72 ± 0.04, SV-HUC-1: 0.13 ± 0.04, *P* < 0.05). SV-HUC-1 was used as a negative control. All the experiments were repeated three times

AID is aberrantly expressed in multiple malignant diseases^17–21^. We measured the expression of AID by western blot and immunohistochemical staining (IHC) in 16 cases of human BUCC tissues. The expression level of AID in BUCC tissue was significantly higher than that of para-BUCC tissue through western blot (BC: 0.98 ± 0.19, PC: 0.16 ± 0.05, *P* < 0.05; Fig. 1a, b). The increased expression of AID in bladder carcinoma tissues was further confirmed by IHC (Fig. 1c, d), and the result is similar with that of western blot (BC: 0.67 ± 0.09, PC: 0.17 ± 0.05, *P* < 0.05). Furthermore, we also measured the expression levels of AID by western blot in T24 and 5637 BUCC cells, together with SV-40-immortalised human urothelial cells (SV-HUC-1) (Fig. 1e, f). The AID expression levels in T24 and 5637 cells were significantly higher than SV-HUC-1 (T24: 0.73 ± 0.07, 5637: 0.72 ± 0.04, SV-HUC-1: 0.13 ± 0.04; *P* < 0.05). Therefore, AID may be involved in the carcinogenesis of BUCC.

### AID knockdown inhibits cell proliferation, migration and invasion and promotes apoptosis of T24 and 5637 cells

**Fig. 2:**
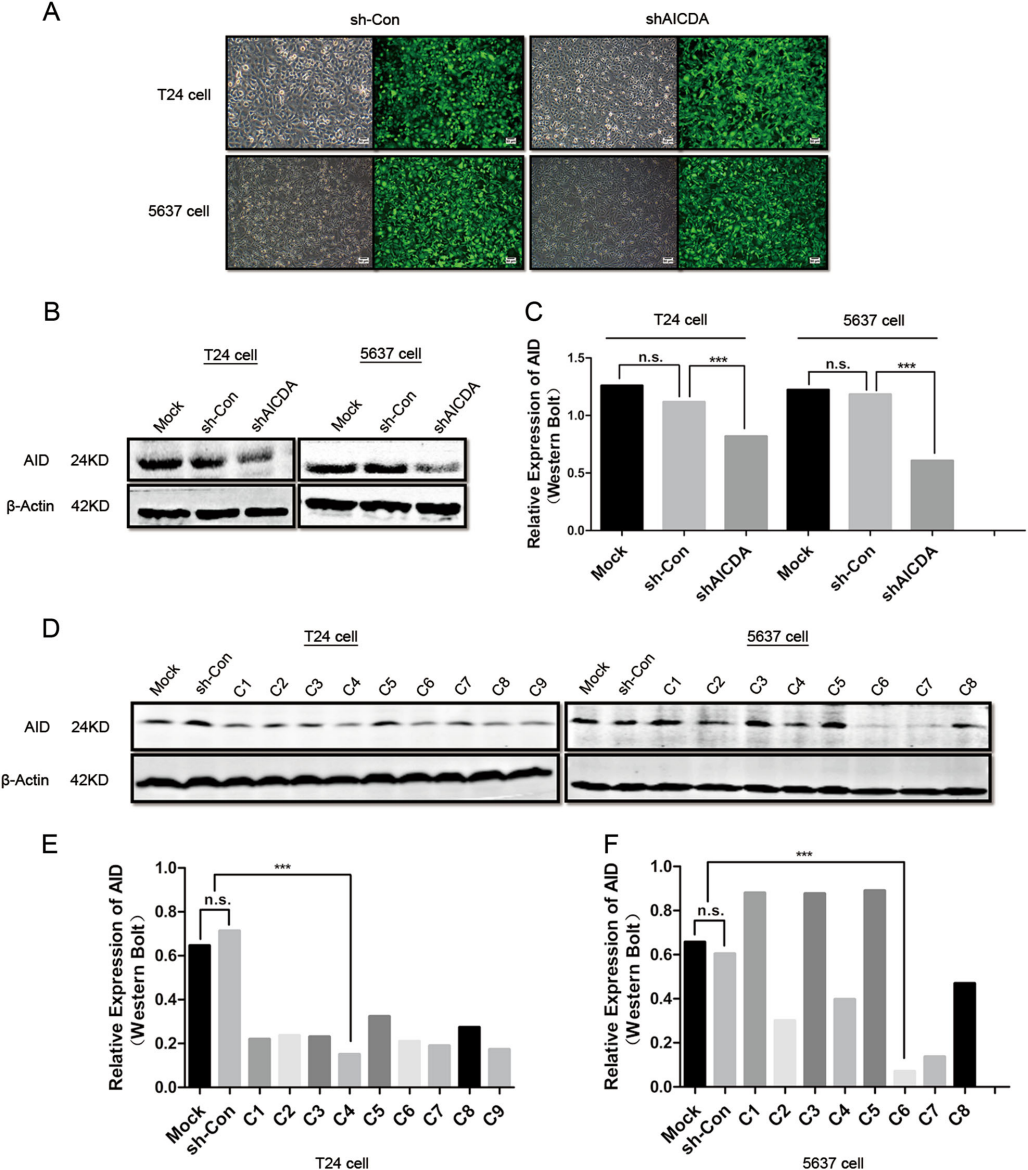

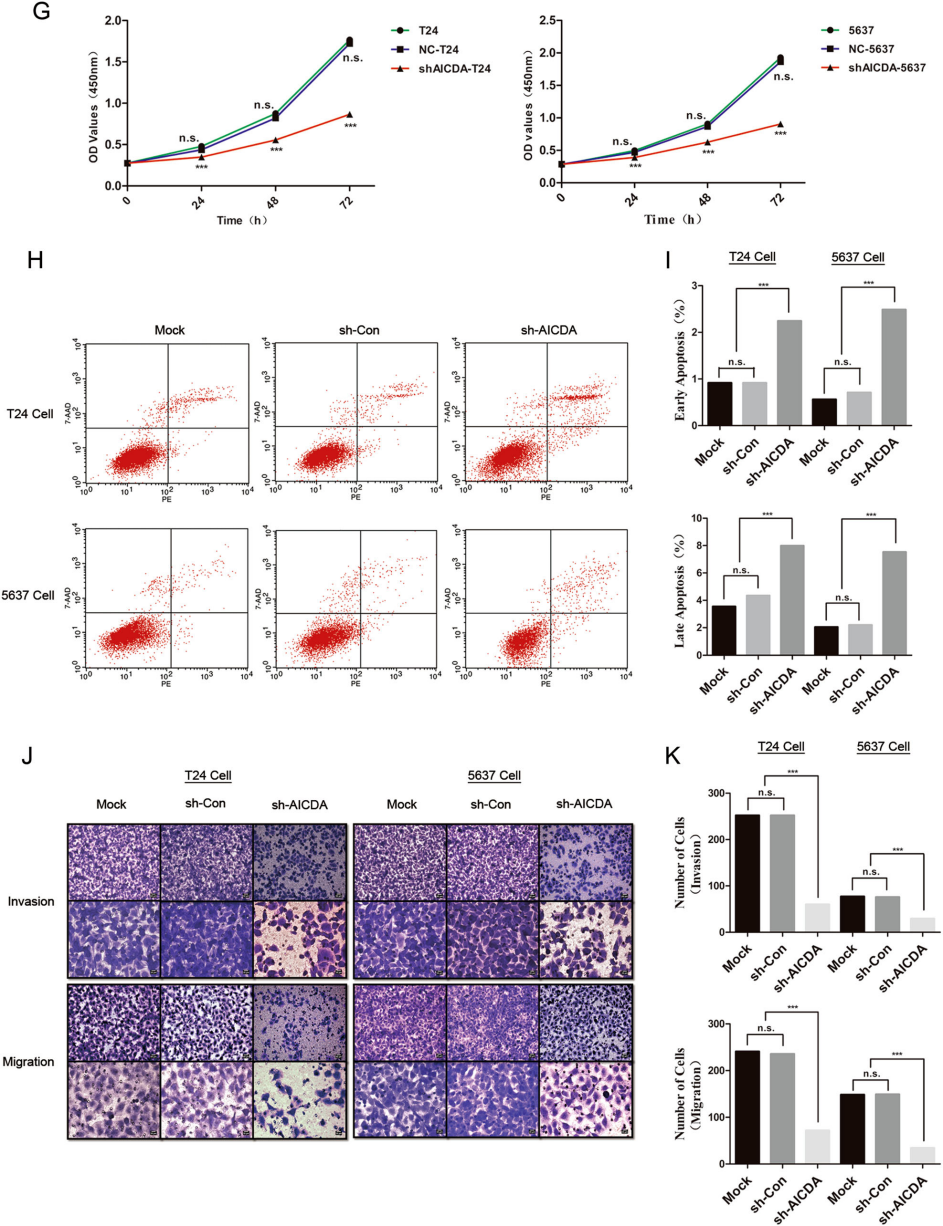
Effect of AID silence on proliferation, invasion, migration and apoptosis in BUCC T24 and 5637 cells. **a** T24 and 5637 cells were transfected with lentivirus-based shRNA-AICDA sequence and negative group transfected with non-sense sequence. Original magnification: **c**, × 200, calibration bar at 50 μm. **b**, **c** Relative expression of AID in T24 and 5637 cells without the screening of monoclonal cells, and suppression ratio of AID of 27% and 50%, respectively. **d**–**f** Expression of AID in T24 and 5637 cells with the screening of monoclonal cells, and suppression ratio improved significantly at 78% and 86%, respectively. **g** CCK-8 assay showed that AID knockdown significantly inhibited T24 and 5637 cell proliferation, with no statistical difference at 0 h but with significant statistical difference between sh-Con and sh-AICDA group at 24, 48 and 72 h (*P* < 0.05). **h**, **i** AID knockdown promoted the apoptosis of T24 and 5637 cells, particularly in the late stage. **j**, **k** AID knockdown suppressed the invasion and migration of T24 and 5637 cells (*P* < 0.05). Original magnification: **j**, × 200 or × 400, calibration bar 50 or 25 μm. All the experiments were repeated three times

Lentivirus-based shRNA was used to suppress the expression of AID in T24 and 5637 cells, with a non-sense sequence being used as a negative control (Fig. 2a). Monoclonal cells were screened to ensure consistent suppression efficiency. Compared with those in sh-Con-T24 and sh-Con-5637 cells, AID expression decreased by 27% and 50% in shAICDA-T24 and shAICDA-5637 cells, respectively (*P* < 0.05; Fig. 2b, c), and the proportion of downregulation was up to 78% and 86% in shAICDA-T24-clone 4 (C4) and shAICDA-5637-clone 6 (C6) cells after screening the monoclonal cells, respectively (*P* < 0.05; Fig. 2d–f). Consequently, the monoclonal cells were used in the following experiments.

Subsequently, we performed Cell Counting Kit-8 (CCK-8) assay to observe the effect of AID silence during T24 and 5637 cell proliferation (Fig. 2g). The OD values were measured at 0, 24, 48 and 72 h; no statistical difference occurred at 0 h, but appeared at 24, 48 and 72 h in both cell lines (*P* < 0.05). The flow cytometry was conducted to investigate the apoptosis rate in T24/sh-Con-T24/shAICDA-T24 and 5637/sh-Con-5637/shAICDA-5637 cells (Fig. 2h, i). AID knockdown significantly increased the percentage of apoptotic cells both in early and late stages, and the difference of late apoptosis was remarkable. To validate the effects of AID on the metastasis in vitro, we performed the Transwell assay with or without Matrigel matrix to evaluate the changes in invasion and migration caused by AID knockdown in T24 and 5637 cells (Fig. 2j, k). Compared with that of T24 and sh-Con-T24 groups, the invasion and migration capability of shAICDA-T24 was significantly suppressed, and similar result was observed in 5637 cell (*P* < 0.05). Thus, downregulation of AID may decrease the proliferation, invasion and migration of T24 and 5637 cells, as well as promote apoptosis.

### AID knockdown inhibits the tumourigenesis and metastasis of T24 cells in vivo

**Fig. 3:**
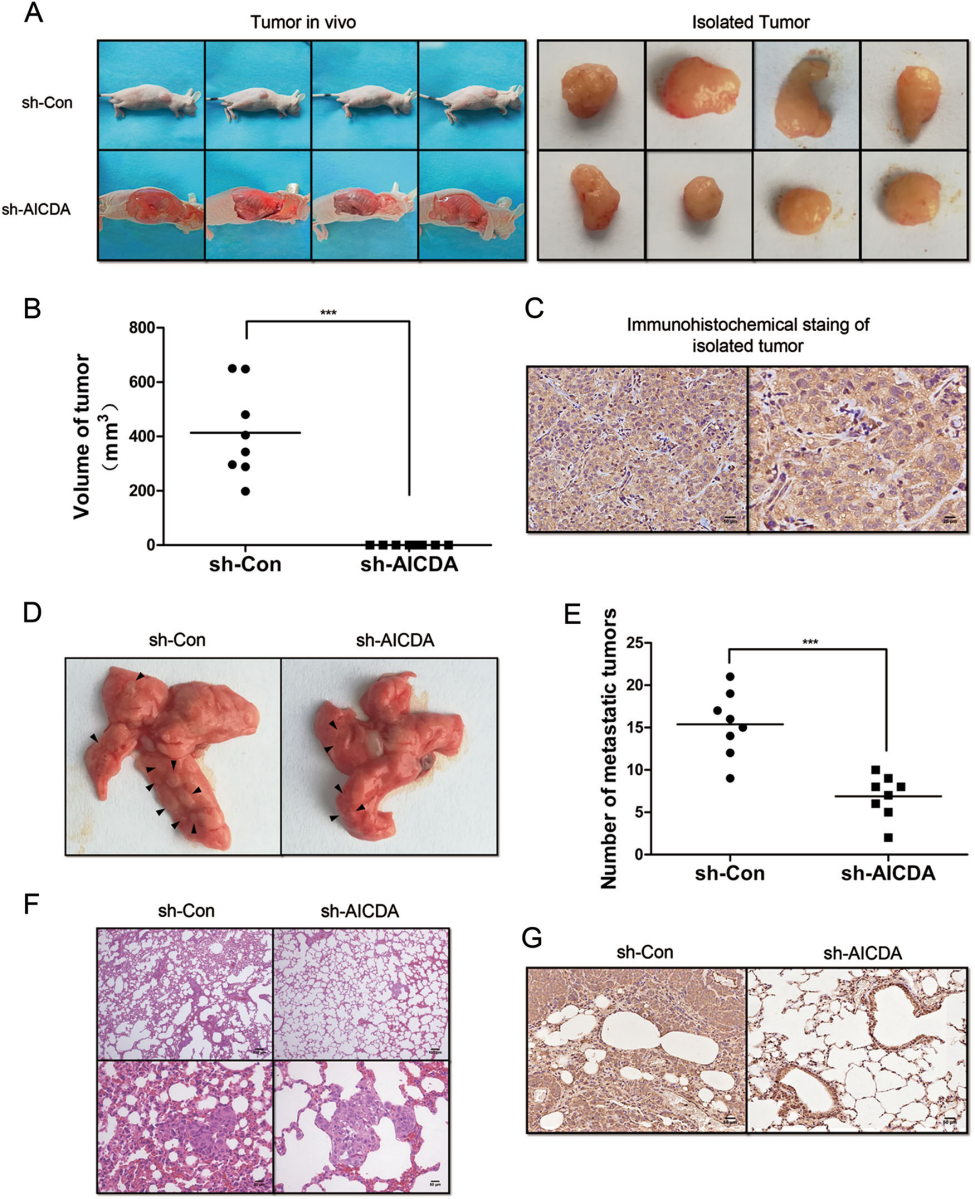
Effects of decreased AID on tumourigenesis and metastasis of T24 cells in vivo. **a**–**c** Suppressed AID expression clearly slowed down tumourigenesis, but no distinct tumour formation in nude mice injected with shAICDA-T24 cells was noted, as described above; *n* = 8. **d**, **e** Interfered AID significantly reduced the lung metastasis of tumour (*P* < 0.05). **f**, **g** Similar result detected by HE and IHC, respectively. Compared with that of nude mice injected with sh-AICDA-T24 cells, the pulmonary alveolus of nude mice in sh-Con group was destroyed more hardly by cancer nest; *n* = 8. Original magnification: **c**, **f**, **g** × 100 or × 200, calibration bar at 100 or 50 μm. All the experiments were repeated three times

To explore the effect of AID knockdown on BUCC in vivo, we established nude murine models injected with sh-Con-T24 or shAICDA-T24 cells to observe the difference of tumourigenesis and metastasis. Tumour volume (in mm^3^) was calculated by the formula, 0.5 × (long diameter) × (short diameter)^2^. The volume of tumour in nude mice injected with sh-Con-T24 cells was 412 ± 147.8 mm^3^ (Fig. 3a, b), and the IHC staining showed that AID expression was intense in the tumour tissue (Fig. 3c). Surprisingly, tumour formation in the nude mice injected with shAICDA-T24 cells was not evident. During the experiment, the injection site of all the nude mice was clearly lifted after injection, illustrating that cells were successfully injected in the subcutaneous layer of nude mice. Thereafter, the nude mice injected with sh-Con-T24 cells gradually formed a distinct tumour. However, the subcutaneous uplift of nude mice injected with shAICDA-T24 cells was apparently shrunk. During the cell culture, we noticed that shAICDA-T24 cells had higher requirements for culture environment than sh-Con-T24 cells. Thus, we speculated that the failure of tumour formation in nude mice injected with shAICDA-T24 cells was caused by nutrient deficiency, inducing the apoptosis of shAICDA-T24 cells. In metastatic experiments, the number of tumours on the lungs of nude mice injected with sh-Con-T24 cells was 15.4 ± 3.6, whereas that in nude mice injected with shAICDA-T24 cells was 6.9 ± 2.4 (Fig. 3d, e), with statistical difference (*P* < 0.05). We also performed haematoxylin–eosin (HE) staining to compare the metastasis of sh-Con-T24 and shAICDA-T24 cells (Fig. 3f). Although the formation of cancer nest was limited in the lung of nude mice injected with shAICDA-T24 cells, the surviving shAICDA-T24 cells strongly expressed AID. This result was further enhanced and required additional AID for tumour formation and metastasis (Fig. 3g).

Therefore, AID knockdown suppressed the tumour formation and metastasis in vivo.

### Identification of AID-related factors via iTRAQ-based proteomic analysis

**Fig. 4:**
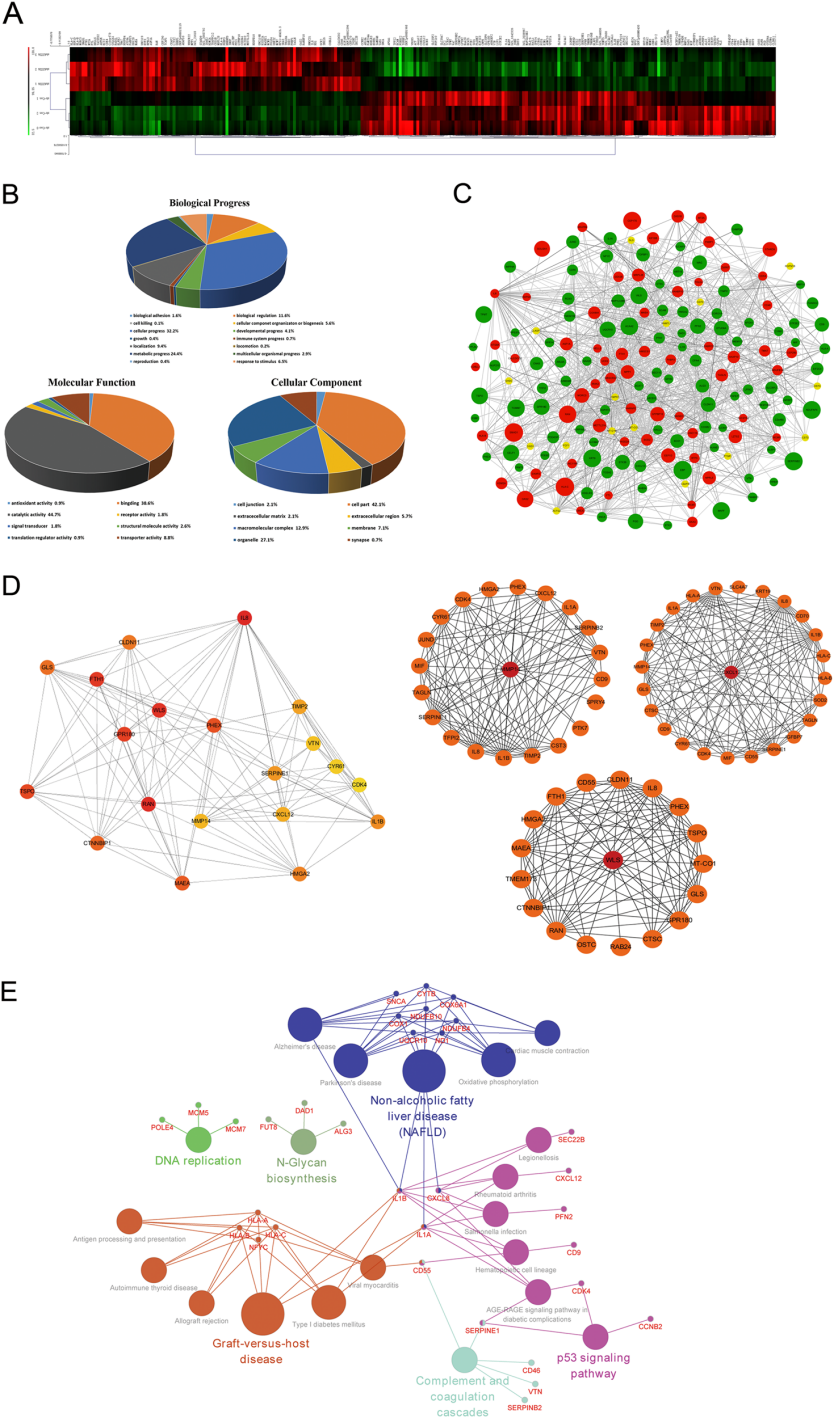
Bioinformatics analysis. **a** Heatmap of altered proteins. A total of 99 and 142 proteins were up- or downregulated with AID silence. **b** Classification of the identified proteins (including biological process, molecular function and cell component) by GO database. Once more than one function was enriched for an altered protein, all the functional annotations were incorporated in the analyses. **c** PPI network established by Cytoscape software. The red and green nodes indicate up- or downregulation, respectively, and the yellow node means the protein is not significantly enriched after AID silence, but closely associated with the altered proteins. The diameter of the node is inversely proportional to the P value. **d** Key factors screened by cytoHubba plug-in and gene annotation. The deeper the colour of the node, the more significant the protein at regulatory position is. Furthermore, according to the previous study of bladder cancer and GO analysis, MMP14, CXCL12 and WLS were finally screened out. **e** The KEGG pathway enrichment of AID-associated proteins. Nodes with larger sizes denote the KEGG pathway terms; the ones with smaller sizes denote the associated proteins. The nodes with more than one colour indicate that they are involved in multiple pathways

AID promotes the development of malignant diseases in multiple ways, including proliferation, invasion and migration, but the mechanism remains unclear. Herein, we performed iTRAQ-based proteomic analysis to identify the AID-related factors by using sh-Con-T24 and shAICDA-T24 cells. Each cell was set up in three subgroups to ensure the reliability of the results. Compared with sh-Con-T24 cells, 99 and 142 proteins were significantly up- or downregulated (> 1.2-fold changes) in shAICDA-T24 cells, respectively (Supplemental Materials 1 and 2). The Multiple Experiment Viewer (MeV) software was employed for clustering analysis (Fig. 4a), and sh-Con-T24 cells were clustered into one class, whereas shAICDA-T24 cells were clustered into the other, indicating that the experimental results were credible. Subsequently, Gene Ontology (GO) analysis was applied to perform the enrichment analysis of function about altered proteins (Fig. 4b). In the biological progress, the changed proteins primarily participated in the cellular progress (32.2%) and the metabolic progress (24.4%). The result of analysis includes biological adhesion, which was related to the metastasis of tumour cells. Regarding MF, 44.7% and 38.6% proteins were involved in catalytic activity and binding, respectively. In the cellular component, the changed proteins were mainly located in the cell part and organelle, and only a few number of proteins participated in cell junction, which was probably associated with biological adhesion.

Furthermore, the Cytoscape software was used to establish the protein–protein interaction (PPI) network (Fig. 4c), the red and green nodes indicate up- or downregulation, respectively, and the yellow node indicates that the protein is not significantly enriched after AID silence, but is closely associated with the altered proteins. Additionally, the large node indicates small *P*-value. Considering the complexity of the network, cytoHubaa plug-in unit was used to realise the dimensionality reduction of network through MCC algorithm (MCC(v) = ∑C ∈ S(v) (|C|−1)!, V denotes random nodes) to identify the proteins in the regulatory status^27^, and top 20 proteins were screened out (Fig. 4d, Supplemental Material 3).

Independent pathway was enriched through Kyoto Encyclopaedia of Genes and Genomes (KEGG). Although the enrichment results were limited, the tumour-related P53 pathway was significantly enriched. This result is consistent with previous reports about AID causing tumourigenesis through the P53 pathway (Supplemental Materials 4 and 5).

Combined with the analysis and the report of BUCC-related factors, MMP14, WLS/GPR177 and CXCL12/SDF-1 were selected to explore the mechanism of AID in promoting the metastasis of BUCC cells (Fig. 4e). Furthermore, MMP14 was also screened out by the gene set enrichment analysis of oncogene (Supplemental Materials 6 and 7). According to the results of proteomic analysis, the downregulation of AID inducing the expression of massive tumour-associated factors changed, consistent with the results of experiment described above. Additionally, MMP14, WLS/GPR177 and CXCL12/SDF-1 were considered as the key factors located in the downstream of AID in BUCC.

### 5-Aza-2’-deoxycytidine antagonises the downregulation of MMP14 and the invasiveness of BUCC cell caused by AID silence

**Fig. 5:**
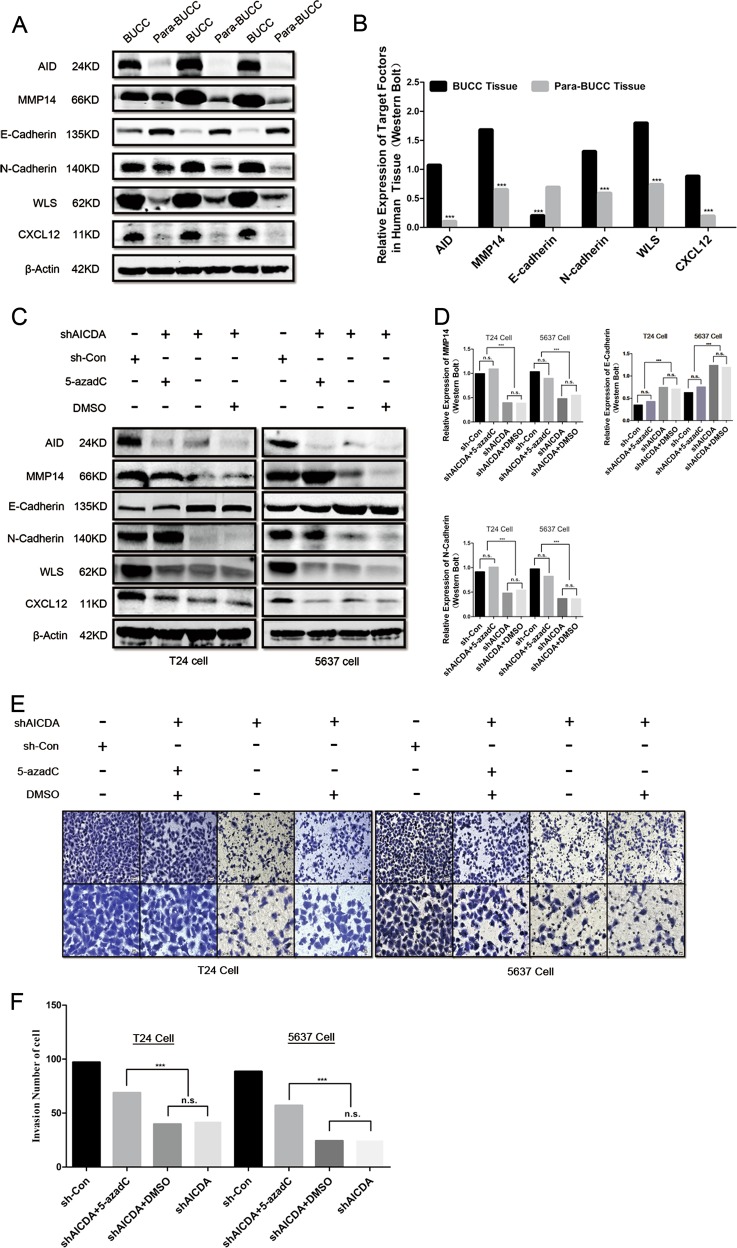
5-azadC promotes the expression of MMP14 and upregulates the invasion of T24 and 5637 cells. **a**, **b** Relative expression of AID, MMP14, E-cadherin, N-cadherin, WLS and CXCL12 in the human BUCC and pare-BUCC tissues. As expected, MMP14, CXCL12 and WLS were significantly highly expressed in human BUCC tissues and relatively less expressed in para-BUCC tissues. Furthermore, pro-EMT factor N-cadherin was also upregulated in BUCC tissues, and the relative expression of E-cadherin was down-regulated—the hallmark of EMT. **c**, **d** Expression of MMP14 in T24 and 5637 cells was significantly recovered after treated with 5-azadC (*P* < 0.05), whereas the expression of CXCL12 and WLS remained unchanged. Moreover, the relative expression of N-cadherin and E-cadherin was up- and downregulated, respectively, when treated with 5-azadC. These results indicated that 5-azadC antagonised AID silencing-induced suppression of invasiveness in T24 and 5637 cell. **e**, **f** Transwell assay of T24 and 5637 cells. The invasiveness of shAICDA-T24 and shAICDA-5637 cells was recovered with the treatment of 5-azadC (*P* < 0.05). Original magnification: **e**, × 200 or × 400, calibration bar at 50 or 25 μm. All the experiments were repeated three times

We first measured the expression of factors described above in human BUCC samples. AID, MMP14, WLS/GPR177, CXCL12/SDF-1 and N-cadherin were highly expressed, and E-cadherin was at a lowexpression level in BUCC tissues compared with those in para-BUCC tissues (Fig. 5a, b).

AID induces demethylation to regulate the expression of genes^4^. If AID silence suppresses the expression of MMP14, WLS/GPR177 and CXCL12/SDF-1 due to demethylation deficiency of these genes, then its expression level should be recovered by treating with the DNA demethylation reagent, 5-aza-2’-deoxycytidine (5-azadC). We measured the expression of AID, MMP14, WLS/GPR177 and CXCL12/SDF-1, as well as that of EMT-related proteins E-cadherin and N-cadherin. Treatment with 5-azadC significantly improved the expression of MMP14 in T24 and 5637 cells (*P* < 0.05), whereas the same phenomenon did not appear among other factors, except E-cadherin and N-cadherin (Fig. 5c, d).

Furthermore, Transwell assay was performed to observe the effect of 5-azadC on the invasion in T24 and 5637 cells. The result shows that 5-azadC recovered invasion ability in both T24 and 5637 cells (Fig. 5e, f).

Therefore, AID may promote the expression of MMP14 through methylation, and the change in E-cadherin and N-cadherin expression levels suggests that AID participates in EMT, regardless of its origin, either from AID directly or from the AID-MMP14 axis.

## Discussion

Since the discovery of AID by Hojo et al.^28^, AID has been studied widely by scholars in different subject areas because of its character in GBCs. Aberrant expression of AID is involved in tumourigenesis and development. Gene mutation is an important mechanism for tumourigenesis, and the imbalance of DNA repair and mutation facilitates the occurrence of tumour. AID-induced point mutation and DNA double-strand break enhance the accumulation of DNA replication errors to promote malignant transformation of cells. Furthermore, AID strengthens tumour progression by demethylation^29^. To date, the study on AID is multitudinous. However, the mechanism of AID promoting tumour progression is not completely clear, especially in BUCC. In this research, we have revealed the AID-related progression factors in BUCC and their possible regulatory mechanisms.

AID is a versatile enzyme consisting of 198 amino acids, mainly expressed in B lymphocyte. However, AID is also aberrantly expressed in non-lymphocytes pathologically. In our study, western blot was used to detect the expression of AID in T24, 5637 and SV-HUC-1 cells, consistent with the results of previous studies on other malignant diseases. AID was also significantly upregulated in T24 and 5637 cells and barely detected in SV-HUC-1 cells. In a previous study, IgG reinforces the development of BUCC in vitro. Furthermore, Thomas Seidl et al. have reported that AID increases the expression of IgG in the cell surface of B cells^30^. However, B cells are no longer the only cell that produces immunoglobulin, but also tumour cells^31^. Hence, AID may function in BUCC cells, but not in human bladder cells. Indeed, we suppressed the expression of AID in T24 and 5637 cells by lentivirus-based shRNA sequence. Decrease in proliferation, invasion and migration and increase in apoptosis were subsequently observed.

In this exploration, we performed an iTRAQ-based proteomics approach to identify AID-related progression factors in AID-silenced T24 cells. Surprisingly, according to available literature, all target factors including MMP14, WLS/GPR177 and CXCL12/SDF-1, are linked to BUCC^32–34^, but the relationship between AID and the factors MMP14, WLS/GPR177 and CXCL12/SDF-1 is unclear. MMP14 is a membrane-type matrix metalloproteinase and is expressed at a low concentration physiologically, except during the stage of embryogenesis^35^. However, similar with AID, MMP14 is also upregulated during inflammation and participates in the process of cancer progression, especially EMT^36,37^. TIMP-2, known as a strong inhibitor of MMP14, unexpectedly, is also downregulated in AID-silenced T24 cells, according to the result of proteomics analysis. This inconsistent phenomenon may explain in this way: MMP14 that catalyses the degradation of the extracellular matrix requires splicing at the optimum site of a triplet of MMP14-proMMP2-TIMP2 to activate MMP2, and the high concentration of TIMP-2 inhibits this process, but the effect of a low concentration of TIMP-2 is opposite^38,39^. Thus, we speculate that a cancer cell downregulates TIMP-2 expression to compensate the insufficiency of hydrolytic collagen induced by AID deficiency. WLS is a kind of the transmembrane transporter that transports Wnt protein produced by the endoplasmic reticulum to the cytomembrane, playing a critical role in the activation of the classical Wnt/β-catenin pathway^40^. The overexpression of WLS strongly promotes the progression of cell cycle of T24 cells^33^. A number of PTK7 in the non-classical Wnt signalling pathway is also downregulated in AID-silenced T24 cells, with statistical significance. In addition, Prebet et al. found that PTK7 reinforces the migration and drug resistance of leukaemic cells^41^. Furthermore, the Wnt signalling pathway is involved in EMT as well through the upregulation of p-GSK3β, β-catenin, c-Myc and cyclin D1 and decreased the expression of GSK3β and p-β-catenin^42^. CXCL12 is a pro-inflammation factor participating in various biological processes under pathological and non-pathological stages. Moreover, CXCL12 promotes the expression of VEGF by activating PI3K/AKT to increase the angiogenesis of glioblastoma^43^. CXCL12 can also be upregulated by phosphorylated Stat3 to strengthen the invasiveness of T24 cells^44^, Coincidentally, Stat3 can also induce the expression of AID^45^. Interestingly, AID, MMP14, WLS and CXCL12 are all associated with embryonic development, EMT and stimulation of inflammation. However, 5-azadC did not recover the expression levels of WLS and CXCL12, and the regulation mechanism between AID and WLS/GPR177 or CXCL12/SDF-1 in BUCC remains unclear. Nonetheless, the functions of these two factors in BUCC indicated that a potential regulation mechanism may potentially exist between them, but such mechanism requires further exploration.

In conclusion, AID knockdown inhibits cell proliferation, migration and invasion and promotes apoptosis in T24 and 5637 cells. Furthermore, AID promotes the expression of MMP14 through demethylation. According to the previous studies, MMP14 facilitates the EMT in many malignant diseases, and the expression of N-cadherin is probably regulated by AID directly or through AID-MMP14 axis, or both. Nevertheless, the upregulation of MMP14 promoted the invasiveness of T24 and 5637 cells, and MMP14 was regulated by AID through demethylation. Although 5-azadC did not improve the expression of WLS/GPR177 and CXCL12/SDF-1, AID knockdown decreased their expression. The same result was observed in human BUCC tissue. Therefore, AID may promote the progression of BUCC through MMP14, WLS/GPR177 and CXCL12/SDF-1, especially during metastasis. Hence, AID is a potential clinical therapeutic target for BUCC.

## Materials and methods

### Cell line and cell culture

The human BUCC cell lines T24, 5637 and SV-HUC-1 were obtained from ASY Biotechnology Ltd., Corp. (Wuhan, China). T24 and 5637 cells were cultured in 90% RPMI 1640 Medium (cat. no. C11875500BT, Life Technologies, Gibco, USA) containing 10% foetal bovine serum (FBS) (cat. no. C10010500, Life Technologies, Gibco, USA) and 1% penicillin–streptomycin (Gibco, USA). Cell was maintained at 37 °C in a humidified incubator with 5% CO_2_.

### Patient selection and tissue preparation

Sixteen men who had been diagnosed with BUCC between January 2016 and December 2017 in Renmin Hospital of Wuhan University were enrolled. Cancer tissue specimens and adjacent tissue specimens identified by experienced pathologist were embedded in liquid nitrogen or paraffin and then subjected to western blot or IHC, respectively. This work was performed in accordance with the Code of Ethics of the World Medical Association and approved by the ethics committee of Renmin Hospital of Wuhan University. Informed consent was obtained for the experimentation with human subjects.

### Generation of AID-specific shRNA-expressing T24 and 5637 cells and selection of stably infected monoclonal colonies derived from T24 and 5637 cells

The AID-specific shRNA sequence was 5′-TGACTTACGAGACGCATTT-3′, and the nonspecific negative control sequence was 5′-TTCTCCGAACGTGTCACGT-3′. Both sequences were designed by GeneChem Corp. (Shanghai, China). Both of them were labelled with green fluorescent protein. The shRNA lentiviral expression vector consisting of the AID-specific or nonspecific sequence was designed and subsequently packaged in lentiviral particles, which were isolated from HEK293T cells (GeneChem Corp., Shanghai, China). The lentiviral particles were then utilised to infect T24 and 5637 cells according to the vendor’s recommendations. T24 or 5637 cells at the concentration of 1000 cells were planted in a 96-well plate containing 200 μl of complete media. After 24 h incubation, the media was replaced with fresh media. Lentiviral particles and polybrene were then added to the plate at a final multiplicity of infection (MOI) of 100. After 12 h infection, the media were replaced with fresh media. After further culturing for 72 h, the cells were digested with trypsin and washed twice with PBS for subsequent suspension in fresh media. The cell concentration was counted, and 100 cells were then transferred to 10 ml of RPMI 1640 media supplemented with 20% FBS in a 100 mm dish (Corning). The cells were maintained in the humidified incubator with 5% CO_2_. After ~20 days, when monoclonal colonies derived from T24 or 5637 cells emerged and could be visible by naked eyes, monoclonal colonies expressing GRP among them were transferred to 24-well plate individually, with media containing with 20% FBS. When the confluences reached to 90%, the monoclonal colonies were transferred to six-well plates filled with fresh complete media for subsequent characterisation.

### Western blot assay

Cells were digested with trypsin and washed twice with PBS, then suspended in radio immunoprecipitation assay buffer (Beyotime Biotechnology, Shanghai, China), mixed with protease inhibitors phenylmethanesulfonyl fluoride (Beyotime Biotechnology) and another protease inhibitor, cocktail (Servicebio Corporation, Wuhan, China), on ice for at least 30 min. After lysing, cell lysates were further crushed using ultrasonic cell crusher prior to the centrifugation in a microcentrifuge at 12,000 g for 10 min at 4 °C and the collection of supernatant. Bicinchoninic acid (BCA) protein assay kit (Beyotime Biotechnology) assay was performed to determine the protein concentration. Then, the supernatant was mixed with 1/4 volume of 5 × sodium dodecyl sulphate–polyacrylamide gel electrophoresis (SDS-PAGE) sample loading buffer (Beyotime Biotechnology). The protein-loading buffer mixtures were boiled at 95 °C for 5 min to denature proteins. Equivalent amount (30 μg) of proteins for cell samples was loaded into wells of a 12% SDS-PAGE gel (Biotechwell, Shanghai, China), along with molecular weight marker (cat. no. 26617, Page Ruler Prestained Protein Ladder, Thermo). Electrophoresis was performed to separate proteins prior to transferring them from the gel to polyvinylidene difluoride (PVDF) membranes (cat. no. IPFL00010, Millipore, USA). The PVDF membranes were blocked for 1 h with 5% non-fat milk (cat. no. 232100, BD Biosciences, USA) at room temperature and subsequently incubated with primary antibodies at 4 °C overnight. Secondary antibodies were then incubated for 1.5 h at room temperature. After rinsed thoroughly within TBST, protein bands were scanned and visualised using Odyssey infrared imaging system (LI-COR Biosciences, USA). Experiments were repeated three times and analysed by image J software.

### Immunohistochemistry

The expression level of AID in tissue specimens was measured by IHC method in accordance with IHC protocol developed by R&D Systems (https://www.rndsystems.com). Briefly, tissue sections were deparaffinised with xylene and alcohol and then rehydrated by buffer solution. Endogenous peroxidase activity was quenched by using a peroxidase blocking reagent.

### Cell proliferation assay

Cell proliferation assay was performed utilising the CCK-8 in accordance with the manufacturer’s instructions (cat. no. CK04, Dojindo, Japan). Briefly, T24 and 5637-derived NC cells and shAICDA monoclonal colonies were inoculated in a 96-well plate to investigate the effect of AID on cell proliferation. At time points of 0, 24, 48 and 72 h, 10 μl of WST reagents were added per well, and the cells were incubated for 60 min. Absorbance was then measured at 450 nm by using a multilabel counter (Perkin Elmer, Singapore).

### Cell migration and invasion assays

Migration assay was performed using 24-well Transwell plates (cat. no. 3422, Corning, USA) with the bottoms of chambers lacking of Matrigel matrix (cat. no. 356234, BD Biosciences, USA), whereas invasion assay was conducted using the Transwell plates coated with Matrigel matrix at a 1:8 dilution ratio. The cells at the concentration of 1 × 10^5^ cells in 100 μl of non-FBS RPMI 1640 medium were seeded in the upper chamber, and 600 μl of RPMI 1640 containing 20% FBS was added in the lower chamber. After incubating for 48 h at 37 °C with 5% CO_2_, cells that migrated or invaded through the bottom of chambers were fixed with 4% paraformaldehyde for 15 min prior to staining with 0.05% crystal violet for 30 min. The number of migration or invasion cells was counted under a microscope.

### Apoptosis assay

Apoptosis assay was performed using phycoerythrin (PE) Annexin V Apoptosis Detection Kit I (cat. no. 559763, BD Pharmingen^™^, USA) following the recommended procedure provided by the manufacturer. Briefly, cells were seeded in six-well plates. After cell attachment, the medium was replaced with FBS-free medium. After starvation for 24 h, the cells were digested with EDTA-free trypsin and neutralised by complete medium. All cells in supernate and in attachment were collected via centrifugation in a microcentrifuge at 2500 rpm for 5 min. Cell pellets were rinsed twice with cold PBS and resuspended in 1× binding buffer. Then, 100 μl of cells at a concentration of 1 × 10^6^ cells/ml were transferred to a 5 -ml culture tube. Furthermore, 5 µl of PE Annexin V and 5 µl of 7-amino-actinomysin (7-AAD) were added into the tube. Then, the cells were gently vortexed and incubated for 15 min at 25 °C in the dark. Finally, the tube was mixed with 400 μl of 1× binding buffer, and the cells were analysed by the flow cytometry (BD FACSCalibur^™^, USA).

### In vivo tumourigenesis and metastasis assays

In tumourigenesis assay, cells were collected via treatment with 0.25% trypsin–EDTA solution and rinsed in Hank’s balanced salt solution (HBSS). After resuspended in HBSS containing 60% Matrigel matrix, cells at a concentration of 5 × 10^6^ cells per 100 μl solution were then injected subcutaneously into male nude athymic BALB/c mice (six-week-old) (*n* = 8). Tumour measurements were performed with calipers at 2-day intervals. Tumour volume (in mm^3^) was calculated by the formula 0.5 × (long diameter) × (short diameter)^2^.

In metastasis assay, cells at a concentration of 2 × 10^6^ cells per 200 μl of HBSS were injected intravenously into the tail vein of nude mice (*n* = 8). All the mice were killed by CO_2_ overdose transiently after 30 days. Afterwards, lung samples were subjected to HE staining. The metastatic tumour area in the field of microscope was calculated using Image-Pro Plus 6.0. All animal experiments were performed in accordance with the National Institutes of Health Guide for the Care and Use of Laboratory animals, as well as approved by the Medical Ethical Committee of the Renmin Hospital of Wuhan University.

### Sample preparation for iTRAQ labelling

A total of 2 × 10^7^ T24-derived sh-Con cells or one monoclonal colony (in which AID expression was most extremely inhibited, *n* = 3) was collected in a tube. The samples were resuspended and thoroughly solubilised by lysis buffer, then sonicated in ice. The samples were bathed in boiling water for 15 min, followed by centrifugation at 14,000 × *g* for 15 min. Debris was discarded, and protein concentration was measured using BCA assay.

A total of 30 μl of protein solution in each sample was mixed with dithiothreitol at a final concentration of 100 mΜ, bathed in boiling water for 5 min, then chilled at room temperature. After adding 200 μl of UA buffer (8 Μ urea, 150 mΜ Tris HCl, pH 8.5), we enriched the proteins by using a 30-kDa centrifugal filter (Sartorius, Germany) at 14,000 × *g* for 15 min; this procedure was repeated twice. Then, 100 μl of iodoacetamide (IAA) buffer (100 mΜ IAA in UA) was added for protein alkylation prior to the vortex for 1 min. After being incubated in the dark for 30 min, the samples were centrifuged at 14, 000 × *g* for 15 min. Afterwards, 100 μl of UA buffer was added and then centrifuged at 14,000 × *g* for 15 min; this process was repeated twice. Furthermore, 100 μl of diluted dissolution buffer (AB SCIEX, USA) was added 10 times in the samples and centrifuged at 14,000 × *g* for 15 min, and then repeated twice. The samples were added with 40 μl of trypsin buffer (4 μg of trypsin in 40 μl of dissolution buffer) and then vortexed for 1 min. After standing for 16 h, the samples were centrifuged again at 14,000 × *g* for 15 min for filtrate. Finally, after the addition of 40 μl of dissolution buffer with dilution for 10 times, the samples were centrifuged at 14,000 × *g* for 15 min. Quantitation of peptides was performed using Nano Drop 2000 (Thermo Scientific, USA).

#### iTRAQ labelling and high-pH RP fractionation

The peptides at 100 μg extracted from each sample were labelled using iTRAQ reagents (AB SCIEX, USA) following the manufacturer’s protocol. The labelled peptides were mixed, and then, chromatography was conducted with Agilent 1260 Infinity II Liquid Chromatography System (Agilent, USA). Labelled peptide mixtures were reconstituted within high-pH RP buffer A (10 mΜ HCOONH_4_, 5% acetonitrile, pH 10.0) and loaded by a manual injector onto a 4.6 mm × 100 mm X Bridge Peptide BEH C18 column (Thermo Scientific, USA). The peptides were eluted at a flow rate of 1 ml/min. The gradient condition was 0% high-pH RP buffer B (10 mΜ HCOONH4, 85% acetonitrile, pH 10.0) for 5 min, 0–7% buffer B for 25 min, 7–40% buffer B for 30 min, 40–100% buffer B for 65 min, and 100% buffer B for 70 min. The absorbance value at 214 nm was detected, and fractions were collected at intervals of 1 min, amounting to 36 fractions. Those fractions were pooled for each sample and lyophilised by vacuum centrifugation. Fractions for each sample were then resuspended in 0.1% formic acid (FA) (Buffer A of the mobile phrase for LC-MS/MS).

#### LC-MS/MS analysis, protein identification and quantification

The sample was separated using EASY-nLC 1200 Liquid Chromatography System (Thermo Scientific, USA) and loaded onto 50 µm × 15 cm Acclaim PepMap RSLC column (Thermo Scientific, USA) at a flow rate of 300 nl/min. The gradient condition was 0–6% buffer B (0.1% FA, 80% acetonitrile) for 5 min, 6–28% buffer B for 45 min, 28%–38% buffer B for 50 min, 38–100% buffer B for 50 min, and held in 100% buffer B for 60 min. After separation, the sample was detected using Q Exactive Plus mass spectrometer (Thermo Scientific, USA). The mass over charge (m/z) range of the precursor ion was set from 350 to 1850 in the MS scan, while MS spectra were obtained at 70,000 resolution. Raw MS data were identified using Mascot 2.5. The parameters used in protein identification when using Mascot 2.5 were as follows: 2 as max missed cleavages, ± 20 ppm as precursor mass tolerance, 0.1 Da as fragment mass tolerance, iTRAQ-8-plex as modification groups from Quan Method, oxidation (M), acetyl (Protein N-term), deamidated (NQ) as dynamic modifications, carbamidomethyl (C) as static modifications, decoy as database pattern and ≤ 0.01 as peptide FDR. In quantification analysis, the protein ratios were calculated as the median of only unique peptides of the protein using Proteome Discoverer 2.1. All those peptide ratios were normalised by the median protein ratio to ensure that the median protein ratio should be 1 after the normalisation.

#### Bioinformatics analysis

The MeV software was used to generate heatmap of the differentially expressed proteins and conduct the cluster analysis. GO functional annotation was used to understand the functional characteristics of the altered proteins. Cytoscape and cytoHubba plugins were employed to establish the PPI network and screen the target factors. Additionally, ClueGo software was utilised to establish the KEGG pathway analysis.

### Statistical analysis

All experiments were performed in at least three biological replicates. Data were shown as mean ± standard deviation (SD). One-way ANOVA or Student’s *t* test was applied for the AID-induced phenotype experiments in vitro and in vivo by using SPSS 23.0 (IBM, USA). Statistical analyses of bioinformatics were based on built-in methods in databases. The level of statistical significance was defined as *P* < 0.05.

## Supplementary information

supplemental Figure Legend

supplemental material 1

supplemental material 2

supplemental material 3

supplemental material 4

supplemental material 5

supplemental material 6

supplemental material 7

supplemental material 8
