# Supplementary material for: AID modulates carcinogenesis network via DNA demethylation in bladder urothelial cell carcinoma
Source: Cell Death Dis. 2019 Mar 15;10(4):251. doi: 10.1038/s41419-019-1472-x (PMC6420503; doi:10.1038/s41419-019-1472-x)
Supplement: Supplementary file 1 — supplemental Figure Legend [file 41419_2019_1472_MOESM1_ESM.doc]

**Supplemental material 1**

The result of iTRAQ-based proteomics analysis, the 99 and 142 proteins in shAICDA-T24 cells were significantly up- or down-regulated, respectively（≥1.2-fold change）compared to sh-Con-T24 controls.

**Supplemental material 2**

The volcano map of the proteomics analysis, the transverse dotted line means P=0.05, and longitudinal dotted line means fold change is 1.2. Among 6452 proteins identified, there is small a mount of protein with a P<0.05 meanwhile fold change significantly, it was proved that the transfection effect was stable.

**Supplemental material 3**

The top 20 protein of network screened by MCC algorithm.

**Supplemental material 4 and 5**

The detailed date of pathway analysis through Cluego software.

**Supplemental material 6**

The detailed date of identification of oncogene by gene set enrichment analysis (GSEA).

**Supplemental material 7**

The heat map of oncogene identified by GESA.

**Supplemental material 8**

The specific and nonsense sequence including was as follows:

shAICDA1: 5’- TTTCGTACTTTGGGACTTT-3’;

shAICDA2: 5’-TTGGTTATCTTCGCAATAA-3’;

shAICDA3: 5’-TGACTTACGAGACGCATTT -3’;

sh-Con: 5’- TTCTCCGAACGTGTCACGT-3’.

(A and B) The relative expression of AID in T24 and 5637 cells transfected with different specific sequence, the shAICDA3 has the best suppression rate among 3 specific sequence, and there is no significant difference in expression of AID between T24 and sh-Con-T24 cells, 5637, and same result wan also observed in 5637 and sh-Con-5637 cells. (C) Cell counting Kit-8 assay was showed that the proliferation of shAICDA3 group was significant down-regulation compared with the shAICDA1 and shAICDA2 group in both cell lines. (D and E). Comparison of apoptosis rate between T24 and 5637 cell line transfected with different shAICDA sequence, and shAICDA3 which with the lowest AID expression has the highest apoptosis rate, both in early and advanced apoptosis. (F and JG) The transwell assay was showed that T24 and 5637 cells transfected with shAICDA3 sequence has the weakest invasiveness. According to these results, the shAICDA3 sequence was selected to use in following experiments.
