# Supplementary figures and images for: AID modulates carcinogenesis network via DNA demethylation in bladder urothelial cell carcinoma
Source: Cell Death Dis. 2019 Mar 15;10(4):251. doi: 10.1038/s41419-019-1472-x (PMC6420503; doi:10.1038/s41419-019-1472-x)

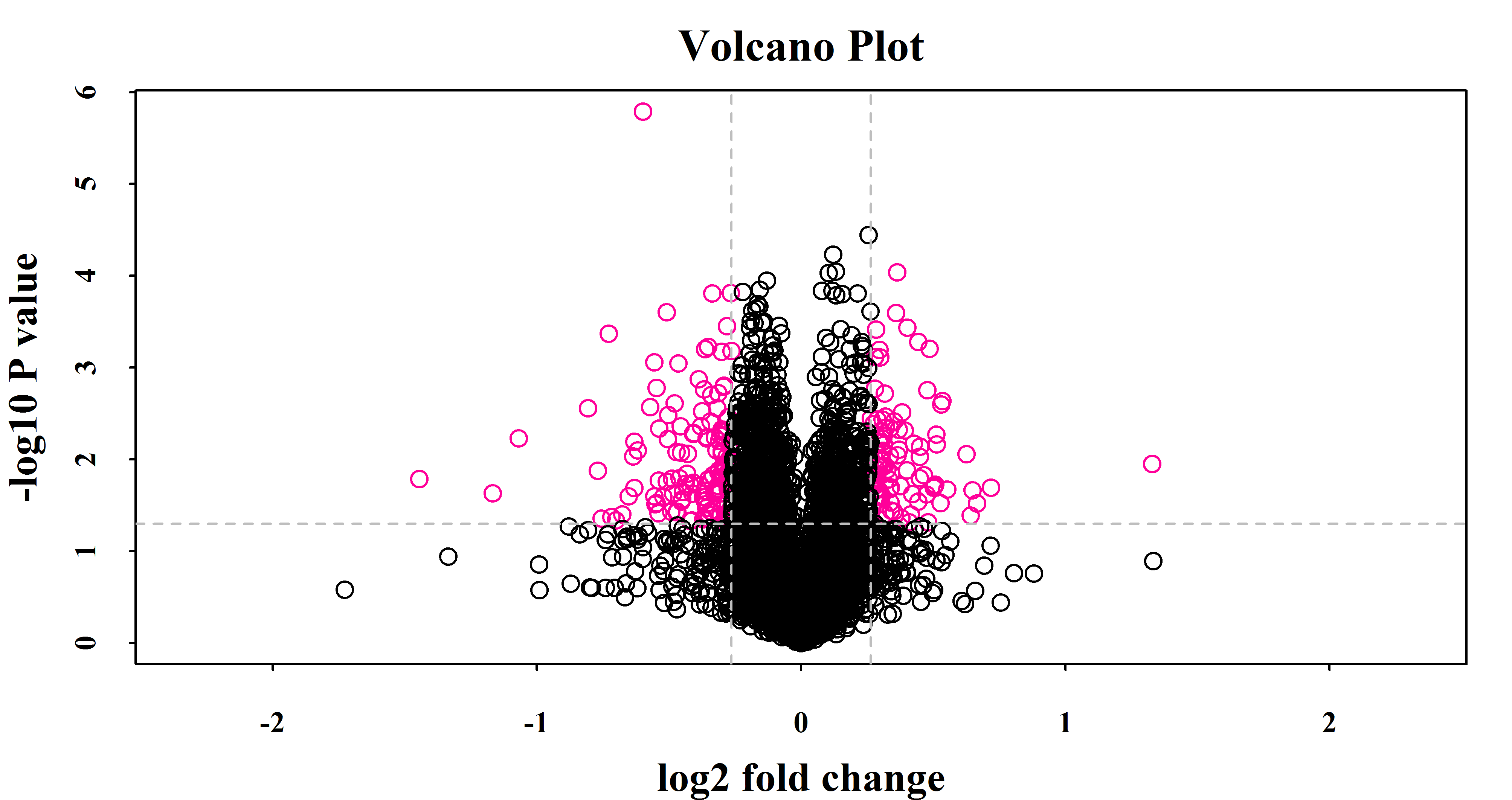

Supplement: Supplementary file 3 — supplemental material 2 [file 41419_2019_1472_MOESM3_ESM.tif]

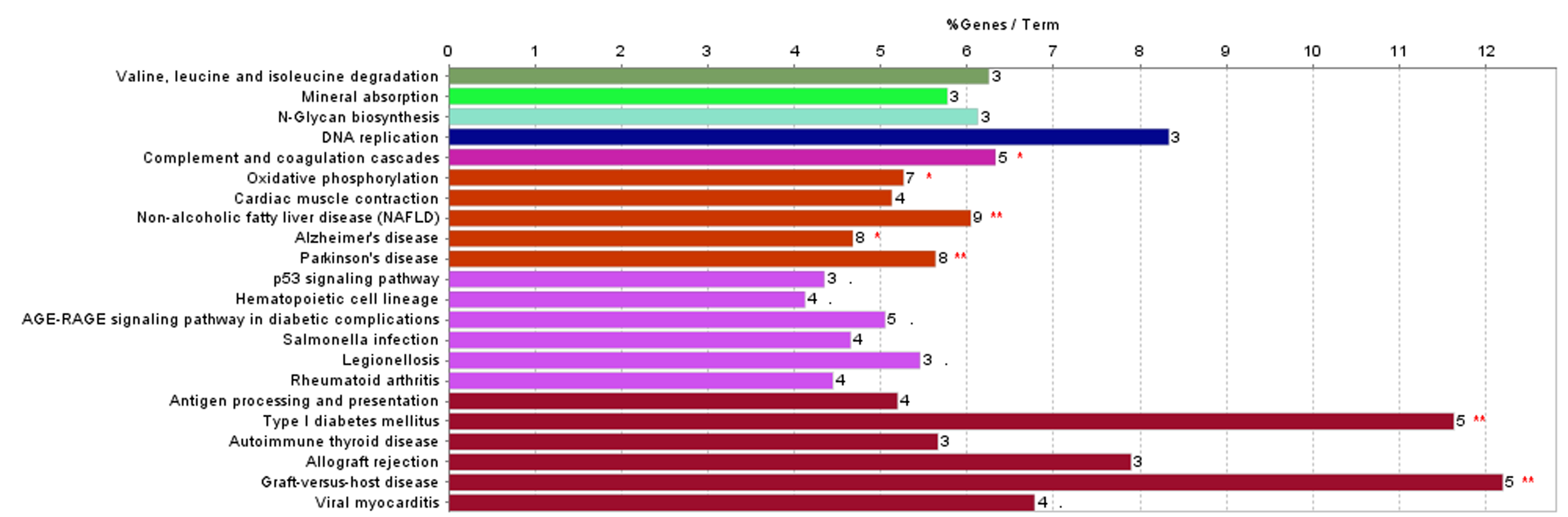

Supplement: Supplementary file 6 — supplemental material 5 [file 41419_2019_1472_MOESM6_ESM.tif]

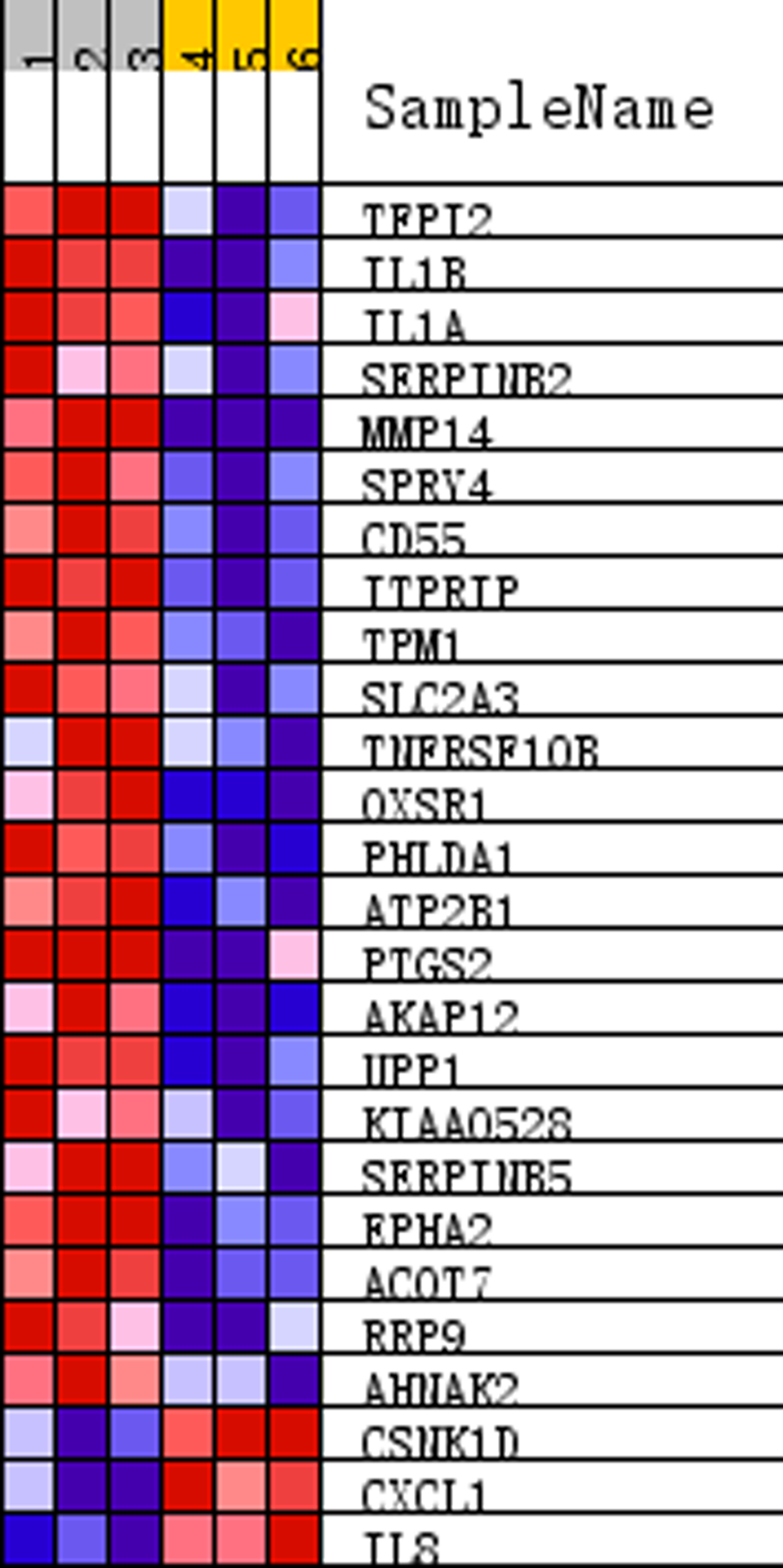

Supplement: Supplementary file 8 — supplemental material 7 [file 41419_2019_1472_MOESM8_ESM.tif]

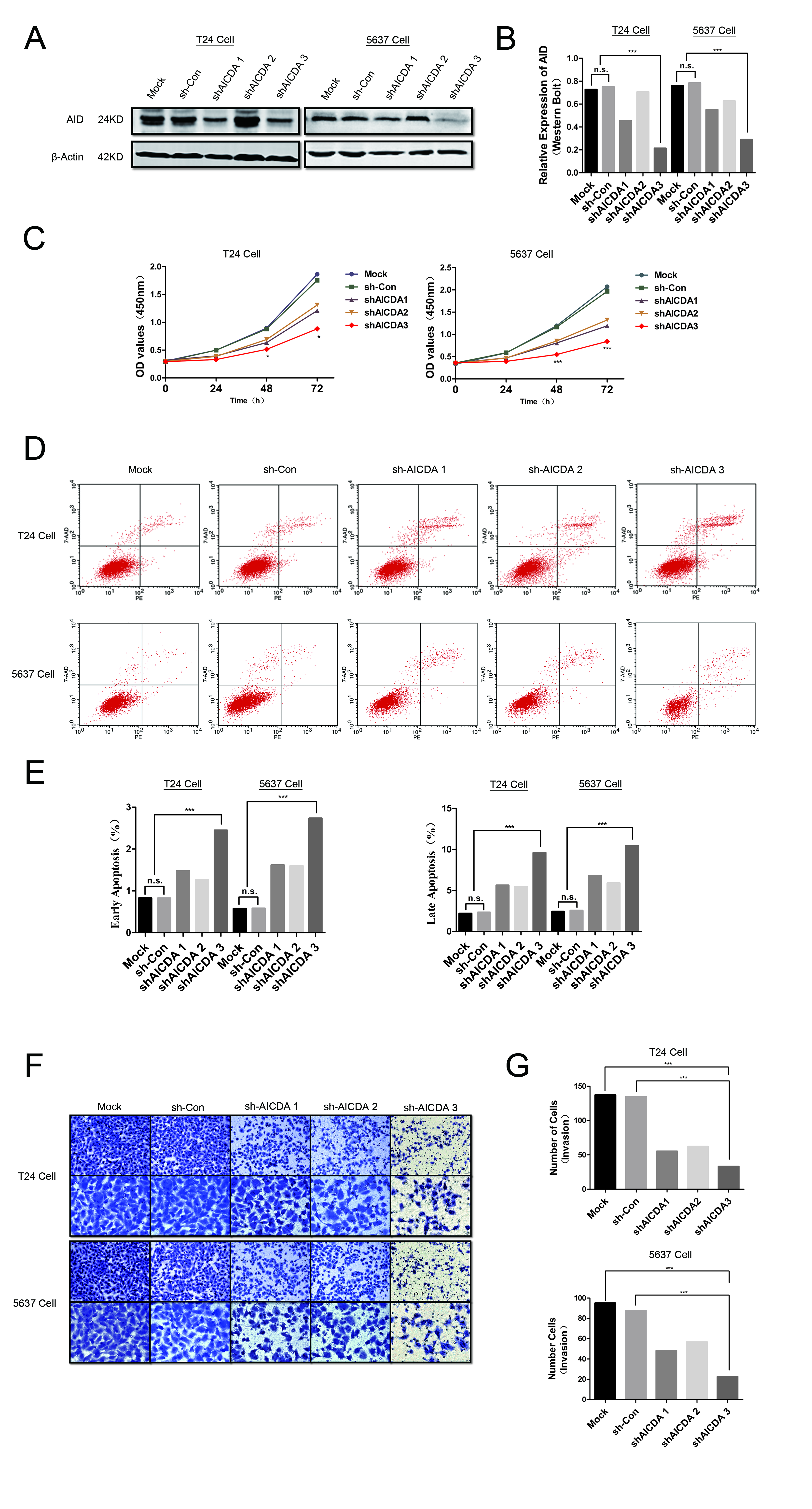

Supplement: Supplementary file 9 — supplemental material 8 [file 41419_2019_1472_MOESM9_ESM.tif]
